# III–V compound semiconductors for mass-produced nano-electronics: theoretical studies on mobility degradation by dislocation

**DOI:** 10.1038/srep22001

**Published:** 2016-02-25

**Authors:** Ji-Hyun Hur, Sanghun Jeon

**Affiliations:** 1 Compound Device Laboratory, Samsung Advanced Institute of Technology, Gyeonggi-Do 446-712, Korea; 2 Department of Applied Physics, Korea University, Sejong 2511, Sejong 339-700, Korea

## Abstract

As silicon-based electronics approach the limit of scaling for increasing the performance and chip density, III–V compound semiconductors have started to attract significant attention owing to their high carrier mobility. However, the mobility benefits of III–V compounds are too easily accepted, ignoring a harmful effect of unavoidable threading dislocations that could fundamentally limit the applicability of these materials in nanometer-scale electronics. In this paper, we present a theoretical model that describes the degradation of carrier mobility by charged dislocations in quantum-confined III–V semiconductor metal oxide field effect transistors (MOSFETs). Based on the results, we conclude that in order for III–V compound MOSFETs to outperform silicon MOSFETs, Fermi level pinning in the channel should be eliminated for yielding carriers with high injection velocity.

Recently, MOSFET scaling has encountered difficulties owing to the limited chip cooling capacity that increasingly necessitates adopting new channel materials with a much higher carrier transport velocity than that of silicon. High carrier velocity allows to reduce power consumption at relatively low operation voltages (<1 V) while maintaining high performance. Thus, materials that exhibit high carrier mobility compared with the bulk silicon have attracted significant attention. The candidate materials can be categorized as three-dimensional and two-dimensional (for example, graphene^1^, MoS_2_^2^, and topological insulators^3^) high mobility materials. Among the three-dimensional high mobility materials, III–V compounds, such as GaAs, InGaAs, and InAs are believed to be the most promising candidates because their bulk electron mobility is more than one order of magnitude larger than that of silicon, while their lattice mismatch with silicon substrate is rather small^4^,^5^,^6^,^7^,^8^,^9^,^10^,^11^,^12^.

Epitaxial growth of III–V compound semiconductors directly on silicon substrates allows compatible integration with silicon technology. However, the biggest problem associated with growing III–V materials on silicon substrates is the difficulty in reducing the density of defects caused by the large lattice mismatches between III–V materials and silicon substrates as well as by their different thermal expansion coefficients. Although the quality of epitaxially grown III–V layers has continued to improve, the measured dislocation densities are typically much higher than 10^6^ cm^−2^ 13,14,15. More critically, for an arbitrarily low average threading dislocation density in an epitaxially grown III–V layer, some MOSFETs in the wafer are likely to exhibit dislocation(s) in the cell’s channel region. For down-scaled (width <20 nm) III–V MOSFET channels with relatively low average threading dislocation density (≤10^8^ cm^−2^), one dislocation is more likely to exist in the channel area. Because mass produced logic circuits have no redundancy in the chip area, only one transistor failure may lead to chip failure. The goal of this work was to determine the extent to which this charged dislocation affects carrier transport characteristics in the down-scaled, quantum-confined MOSFET.

There have been several theoretical studies on the extent to which electron mobility is affected and degraded by charged dislocations in wurtzite GaN^2^,^3^,^4^,^5^,^6^,^7^. However, this effect has not been investigated for III–V semiconductor-based devices; as a matter of fact, the relationship between the extent of charged dislocations in the III–V channel layer and the carrier mobility was only approximately estimated by Hall mobility that was measured in bulk samples with different dislocation densities.

In this paper, we present a model for carrier scattering by charged dislocations in quantum-confined III–V semiconductor MOSFETs. Among the existing III–V materials, we choose In_0.53_Ga_0.47_As as a channel material because In_0.53_Ga_0.47_As is the most popular III–V channel material used in field effect transistors owing to its rather high electron mobility and relatively small lattice mismatch with silicon substrate. Dislocations lined up normal to the carrier transport direction create two main effects. One is the effect of deformation potential that is caused by the atomic displacement of dislocations. Because the deformation potential created by the dislocations is primarily neutral (except for the piezoelectric coupling case), the effect is much lower owing to its shorter range of interaction compared with the other, long range, scattering mechanisms. The second effect is the Coulomb potential perturbation owing to the charges that are trapped at dislocations. Because the Coulomb interaction has much longer range than the deformation potential interaction, the dominant effect arises from the Coulomb interaction between carriers and charged dislocations. The model can be applied to any III–V compound semiconductors materials that have dominant conduction valley at gamma point with almost symmetric effective masses regardless of carrier transport directions. By adjusting materials parameters that our model concerns, the model can be applied semi-universally to any III–V materials with dislocations.

## Results

**Figure 1:**
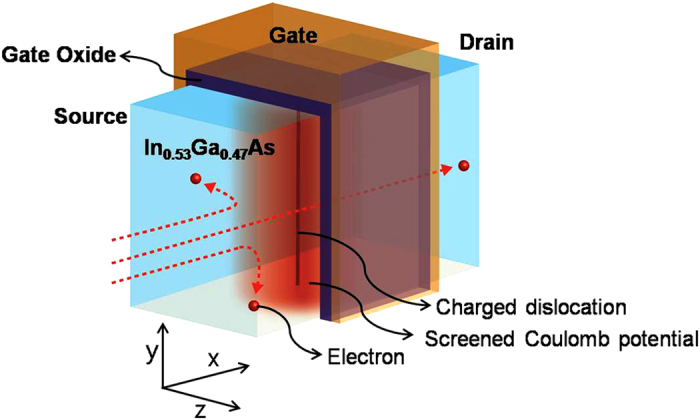
Schematic illustration of the III–V semiconductor channel fin-shaped field effect transistor with a charged dislocation located at center of channel and vertical to the top gate surface.

Quantum-confined (with discrete energy levels) MOSFETs include systems such as fin-shaped channel field effect transistors (FinFETs), channel on-insulator transistors, and quantum well confined high electron mobility transistors (HEMT). Because we are dealing with the MOSFET on-state, the free carrier density is not determined by the neutrality condition^4^ but only by the chemical potential of the channel carriers, which can be controlled by the gate voltage. We further assume in this paper that dislocations are normal to the In_0.53_Ga_0.47_As surface because threading dislocations preferentially line up in the direction of epitaxial growth. Schematic of our model is shown in Fig. 1. Figure 1 illustrates the carrier scattering events in In_0.53_Ga_0.47_As FinFET with a charged dislocation located near the center of the channel that is fully charged, screened by the environmental free carriers, and vertical to the top gate.

It is known that dislocations in arsenides can create acceptor-like defect states, acting as electron and hole recombination centers or electron traps^8^,^9^,^10^,^11^. We assume in this paper that these initially empty acceptor-like traps are filled by one electron per lattice length parameter, at an elevated electron chemical potential relative to the conduction band edge in the channel (transistor on-state). First, we calculate the rate of carrier momentum relaxation caused by the Coulomb interaction with charged dislocations in the on-state FinFET with quantum confinement in the channel width direction (*z*-direction). For this calculation, we assume that the wave-vector *k* of the incident (initial) electrons is in the *x*-direction (channel length direction). On the other hand, the scattered (final) wave-vectors can have components in both *x*- and *z*-directions. We assume that the wave confinement by gate oxide barriers occurs only in the channel width direction (*z*) whereas in the *x*-direction the wavefunction is approximated as a free wave function. Penetrations of the electron wave function into the gate oxide, which can be regarded as an effective confinement width increase, are neglected. Thus, the initial and final wave functions can be simply represented as follows


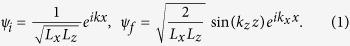


Here, the subscripts *i* and *f* represent the initial and final states, L_x_ and L_z_ are the channel length and width, *k* is the initial wave-vector, and k_x_ and k_z_ are the final wave-vectors in the *x*- and *z*-directions. The wave-vector k_z_ is quantized, and can assume discrete values of n_z_π/L_z_, n_z_ = 1, 2, 3…. The *y* components of the wave-vectors are not considered because scattering owing to the dislocation line charges cannot change the momentum in the line direction. Therefore, the scattering physics in this situation can be treated as a two-dimensional problem^4^.

From the transition rate relation of elastic scattering that results in Fermi’s golden rule and screening effect that considers the Coulomb potential expression in the momentumspace^4^, the dislocation scattering rate (S_dis_) can be written as


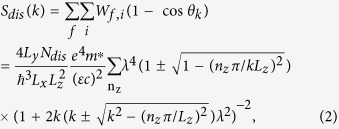


Here, W_f,i_ is the transition rate from the *i* state to the *f* state, θ is the angle between the initial and final wave-vectors, N_dis_ is the number of charged dislocations in the channel, m* is the effective mass of electrons in the channel, ε is the dielectric permittivity of the In_0.53_Ga_0.47_As, *c* is the lattice parameter along theIn_0.53_Ga_0.47_As layer’s growth direction, *e* is the unit charge, n_z_ is a positive odd number including zero, of which the maximal number is determined by the energy conservation relation, and λ is the Debye length (=(εk_B_T/e^2^n_e_)^1^/2). We consider confinement-length dependent effective mass variations originating from non-parabolic band as^13^


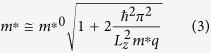


Here, 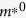
is the bulk effective mass (=0.041 m_0_) in the Γ-valley of In_0.53_Ga_0.47_As. For derivation of Eq. (2), we assumed that the Debye length, which is typically ~1 nm, is smaller than the confinement length (L_z_), allowing us to perform Fourier integration.

The free electron density is a function of the carriers’ Fermi energy and can be represented as a sum over all possible energy states below the Fermi energy,


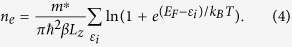


**Figure 2:**
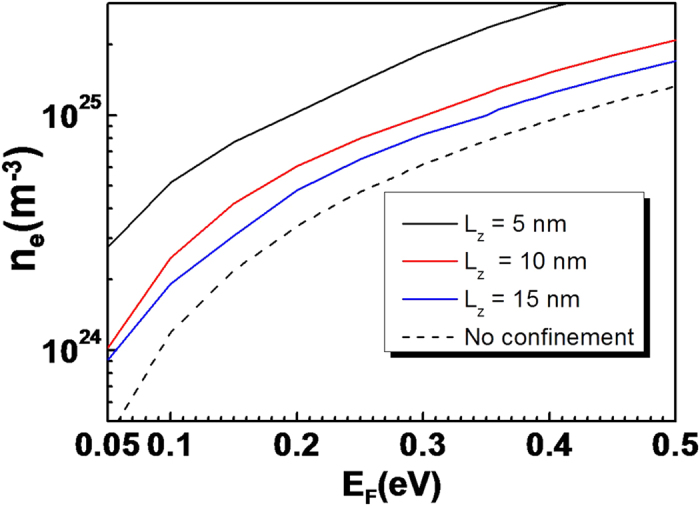
Free electron densities with respect to Fermi energies (0 at conduction band minimum), for several channel widths (L_z_), ranging from 5 nm to 20 nm. For this calculation, the temperature T was room temperature (300 K), L_x_ was 10 nm, L_y_ was 20 nm and the material parameters of In_0.53_Ga_0.47_As were as follows: dielectric constant = 13.56, lattice parameter c = 5.87 Å and m^*0^ = 0.041 m_0_ (at Γ-valley).

Here, ε_i_ is the *i*^th^ possible energy below the Fermi energy (E_F_) in the conduction band quantized in the *z*-direction and β = 1/k_B_T. The obtained free electron densities are shown in Fig. 2 as a function of the Fermi energy for several channel widths (L_z_), for In_0.53_Ga_0.47_As as a channel material. As can be expected from Eq. (4), there is an almost linear relationship between the Fermi energy and free carrier density. The calculated electron volume density is inversely proportional to the channel width, owing to the increasing total volume with increasing channel width. The carrier density approaches the ideal isotropic, infinite three-dimensional carrier density (~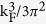) as the channel width approaches infinity, as is shown in Fig. 2 by the dotted line.

Although the electric field in the channel is rather small owing to the low operation voltage for down-scaled devices, there is a non-vanishing electric field in the channel. This electric field and the scattering events determine the Fermi-Dirac distribution functions of the electrons. By solving Boltzmann’s transport equation using relaxation time approximation, which can be justified by noting the elastic nature of the Coulomb scattering^13^, the distribution can be written in terms of the electric field and scattering time as


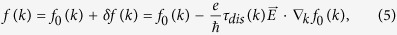


where f_0_(k) is the equilibrium distribution function, E is the electric field in the channel, and τ_dis_(k) is the average scattering time or the mean free time between scattering events, which is equal to S_dis_(k)^−1^. Then, the average carrier velocity can be calculated by assuming k ~ k_x_ as


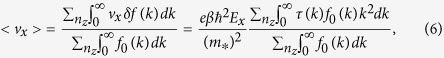


Then, from the relation between the average carrier velocity and mobility (μ_dis_ = <v_x_> /E_x_), the dislocation mobility is finally obtained as^16^


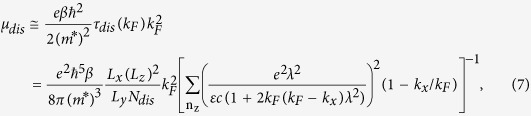


where k_F_ is the Fermi wave-vector and 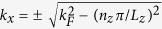.

**Figure 3:**
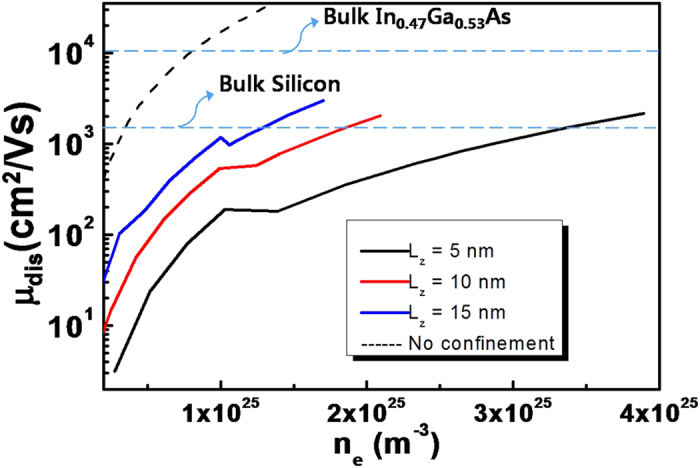
Dislocation mobility values as a function of electron densities for different channel widths (L_z_), ranging from 5 nm to 15 nm. The dislocation mobility without quantum confinement effect, obtained in ref. 4 for the dislocation density of 5 × 10^11^ cm^−2^ is also plotted for comparison. Parameters are the same as in Fig. 2.

Dislocation mobility, obtained from Eq. (7) in terms of the electron densities for different channel widths is plotted in Fig. 3. Here again, the channel material is assumed to be In_0.53_Ga_0.47_As, with detailed material constants listed in the caption of Fig. 3. It can be seen that the dislocation mobility remains much smaller than the bulk lattice mobility of In_0.47_Ga_0.53_As (~10^4^ cm^2^/Vs) and even smaller than that of bulk silicon for most Fermi energies regardless of the channel width. We also plot in this figure that the dislocation mobility calculated for the non-confined system (ref. 4 ) with the dislocation density of 5 × 10^11^ cm^−2^, corresponding to the density of one dislocation in the L_x_ (10 nm) by L_z_ (20 nm) area. In other words, in a quantum confined In_0.53_Ga_0.47_As nano-device, electron density should be larger than ~2 × 10^25^ m^−3^ for L_z_ = 10 nm and larger than ~4 × 10^25^ m^−3^ for L_z_ = 5 nm, for outperforming silicon device with the same structure. This implies not only the need for a high operation voltage for increasing the carrier energy, but also elimination of the Fermi level pinning in the channel. Although the Fermi energy (and injection velocity) of carriers in the channel depends only on the gate oxide and operation voltage, it cannot attain arbitrary values for a fixed gate oxide by adjusting the gate voltages. In general, there exists Fermi level pinning owing to the presence of interface traps. According to the recent reports on the properties of InGaAs/Al_2_O_3_ interface structures (see ref. 14), the Fermi energy is pinned at 0.21–0.35 eV above the conduction band edge. Therefore, it is essential for an III–V nano-device to maintain the channel/oxide interface as clean as possible for gaining comparative advantage over a silicon device, even if the channel contains dislocations. In general, four methods can be used for minimizing the trap density at the channel/oxide interface. The first method is engineering the interface by any treatment process such as cleaning before the gate oxide deposition, adopting interfacial layers, fine control of the deposition process, and post-treatment by annealing, to name a few^16^,^17^,^18^. The second method is changing the crystalline orientation of III–V compounds from 100 to other orientations^19^,^20^,^21^. The third method is fine tuning of atomic fractions of compounds. For example, it was reported that increasing the indium content of InGaAs channel can improve interface conditions^22^. The last method amounts to adopting more suitable (compared with conventional) oxides for specific channel materials^23^,^24^.

In this figure, on the whole, the dislocation mobility is proportional to the electron density which is the opposite to the case of bulk semiconductors with charge neutrality. Because, in MOSFET, carrier density is independently determined by the gate capacitance and the gate voltage, the conventional inverse proportionality between the carrier density and mobility no longer holds for MOSFETs. It is also noticeable in the figure that step-like drops in mobility appear at specific Fermi energies for each channel width which can be explained by increases in the number of available k_z_ (and by an increased number of scattering paths) that results in an increase of the scattering frequency.

**Figure 4:**
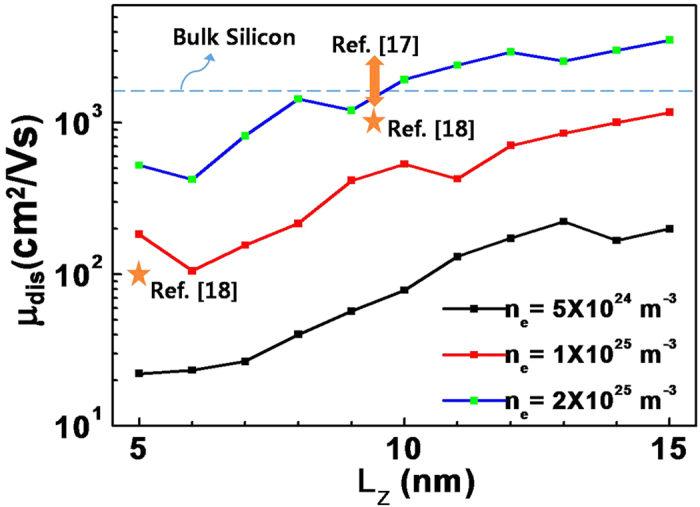
Dislocation mobility values as a function of channel width, for different electron densities. Parameters are the same as in Fig. 2.

The dependence of dislocation mobility on the channel width is shown for different electron densities in Fig. 4. In this figure, we also mark previously reported experimental results of In_0.53_Ga_0.47_As quantum confined MOSFETs^17^,^18^ to estimate the effect of dislocation scattering on devices. From the comparison, we conclude that the dislocation scattering could be the most dominant source of scattering for quantum-confined MOSFETs. The figure shows that there is an approximate (with some ripples originated from quantum confinement effect) linear relationship between the dislocation mobility and channel width. This relationship reflects an effective mass modification by band non-parabolicity and variation in the effective threading dislocation density owing to the cannel area variation. Again, as shown in Fig. 3, mobility is even smaller than that of bulk silicon for most values of the fin width and for electron densities below 1 × 10^25^ m^−3^ for L_z_ of 15 nm or smaller.

The total carrier mobility μ_tot_ is determined by employing Matthiessen’s rule with μ_dis_ and μ_0_ (1/μ_tot_ = 1/μ_0_ + 1/μ_dis_), where μ_0_ is the carrier mobility without dislocations that includes all contributions to the scattering mobility (μ_i_), such as the dopant scattering, interface trap scattering, surface roughness scattering, and phonon scatterings, and is expressed as 1/μ_0_ = ∑_i_1/μ_i_. Thus, if the rate of dislocation scattering is much larger than those of other scattering sources which is believed to be the case for nano-scale channel length, it would govern the carrier transport in the device.

## Discussion

It would be useful to discuss the satellite valley (L, X) conduction in In_0.53_Ga_0.47_As channels. Owing to the relatively small valley separation between the Γ-valley and L/X valleys (0.46 eV for Γ-L and 0.59 eV for Γ-X), there is a non-vanishing probability for electrons to be in the satellite valleys, especially for high electric field applications. If we set the electron density ratio n_i_/n_Γ_ between the *i*-valley and Γ-valley as *f*_*i*_ (0 < *f*_*i*_ < 1) and assume that the effective electron mass in the Γ-valley is much smaller than that in the other valleys, the averaged dislocation mobility can be rewritten as follows^15^


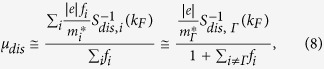


Here, 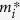 is the electron’s effective mass in the *i*-valley. As can be seen from Eq. (8), the obtained average dislocation mobility can be approximated by the dislocation mobility that only considers the Γ-valley conduction (Eq. (7)) divided by the factor 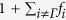. Because we are dealing with down-scaled MOFSETs with lower operation voltage (<0.8 V), the values of f_i_ are probably close to unity; however, a detailed estimation will be reported in the future work.

In summary, we have developed a theoretical model for charged dislocation scatterings in quantum-confined MOSFETs. The model was applied to the In_0.53_Ga_0.47_As channel system that was expected, for the dislocation free case, to exhibit much higher carrier mobility than silicon. The calculated dislocation mobility was even smaller than that of bulk silicon for a wide range of Fermi energies (E_F_ <0.5 eV). From this result, we conclude that a nano-scale III–V compound semiconductor device loses its merits over silicon if it bears inevitable (when fabricated on silicon wafer) dislocation(s). To solve this problem, two preconditions should be met. One is the requirement of high operation voltage for raising the carrier velocity, and the other is a almost perfect match between the gate oxide and III–V channel material for eliminating Fermi level pinning. However, the former is limited by the leakage power concern and the latter remains unresolved. The calculated mobility values were compared with experimentally measured effective mobility values in carrier-confined devices that have all the scattering sources such as the surface roughness scattering, phonon scattering, and interface trap scattering, for estimating the dislocation scattering influence. Based on this comparison, we conclude that dislocation scattering could be the most dominant scattering mechanism in quantum-confined, short channel MOSFETs. This severe degradation of the effective channel mobility would lead to malfunctioning of the transistor cell, eventually resulting in the failure of the entire logic chip.

## Additional Information

**How to cite this article**: Hur, J.-H. and Jeon, S. III–V compound semiconductors for mass-produced nano-electronics: theoretical studies on mobility degradation by dislocation. *Sci. Rep.* **6**, 22001; doi: 10.1038/srep22001 (2016).
